# Inverse Association of the Adequacy and Balance Scores in the Modified Healthy Eating Index with Type 2 Diabetes in Women

**DOI:** 10.3390/nu15071741

**Published:** 2023-04-02

**Authors:** Hye-Jeong Yang, Min-Jung Kim, Haeng-Jeon Hur, Dai-Ja Jang, Byung-Kook Lee, Myung-Sunny Kim, Sunmin Park

**Affiliations:** 1 Food Functionality Research Division, Korea Food Research Institute, Wanju 55365, Republic of Korea; 2 Department of Preventive Medicine, Soonchunhyang University, Asan-si 31538, Republic of Korea; 3 Department of Food Biotechnology, University of Science & Technology, Wanju 55365, Republic of Korea; 4 Department of Food and Nutrition, Obesity/Diabetes Research Center, Hoseo University, Asan-si 31499, Republic of Korea

**Keywords:** health eating index, type 2 diabetes, vitamin C, calcium, noodles

## Abstract

Type 2 diabetes (T2DM) has markedly increased among Asians as their diets and lifestyles become more westernized. We, therefore, investigated the hypothesis that the Korean healthy eating index (KHEI) scores are associated with gender-specific T2DM risk in adults. The hypothesis was tested using the data from the Korea National Health and Nutrition Examination Survey-VI (2013–2017) with a complex sample survey design. Along with the KHEI scores, the modified KHEI (MKHEI) scores for the Korean- (KSD) and Western-style diets (WSD) were used as independent parameters, calculated using a validated semi-quantitative food-frequency questionnaire (SQFFQ). We estimated the association between the KHEI or MKHEI and the T2DM risk using logistic regression after adjusting for T2DM-related covariates. The adults with T2DM were more frequently older men who were less educated, married, on a lower income, and living in rural areas compared to those without T2DM. Not only the fasting serum glucose concentrations but also the waist circumferences and serum triglyceride concentrations were much higher in adults with T2DM than in those without T2DM in both genders. Serum HDL concentrations in the non-T2DM subjects exhibited a greater inverse relationship to serum glucose than in the T2DM group in both genders. Twenty-four-hour recall data revealed that women, but not men, had higher calcium, vitamin C, saturated and monounsaturated fatty acids, retinol, and vitamin B2 intakes than the T2DM group. Furthermore, overall, the KHEI score and the adequacy and balance scores among its components were significantly higher in the non-T2DM group than in the T2DM group, but only in women. The KHEI scores were inversely associated with T2DM only in women. The mixed grain intake score was higher in the non-T2DM than the T2DM group only in men. However, there were no differences between the groups in the MKHEI scores for KSD and WSD. In conclusion, high KHEI scores in the adequacy and balance components might prevent and/or delay T2DM risk, but only in women.

## 1. Introduction

The incidence of type 2 diabetes (T2DM) has been steadily increasing worldwide over the last three decades. The World Health Organization (WHO) reports a four-fold increase in the incidence of adults with T2DM since 1980. In the United States (US), approximately 10.5% of the population has T2DM, with a higher prevalence among certain ethnic and racial groups. In Asia, the prevalence of T2DM has been suddenly on the rise. The prevalence in Korea among adults aged 30 years or older is 15.9% for men and 11.8% for women [1]. In China, the overall prevalence of T2DM has been estimated to be about 13.6% [2]. The incidence of T2DM in South Asia is estimated to increase by more than 150% from 2000 to 2035 [3].

Furthermore, South Asians develop T2DM at a younger age, which occurs in individuals with lower adiposity than Caucasians [4]. Traditionally, the South Asian lifestyle included whole grains and a low-fat diet combined with high physical activity, which contributed to insulin sensitivity and a lower risk of developing T2DM. However, with urbanization, refined grains and fat consumption have increased. The urban lifestyle involves less physical activity, which has led to an increase in the development of insulin resistance. The fat intake of South Asians remains lower than that of Caucasians. Nevertheless, South Asians rapidly shift from an insulin-resistant state to frank T2DM due to their limited insulin-secretory capacity and smaller β-cell mass compared to Caucasians. Therefore, the rapid and sudden increase in the prevalence of T2DM, which has reached epidemic proportions, especially in South Asians, is related to lifestyle changes that are associated with urbanization [3]. Therefore, lifestyle-related guidelines for Asians are necessary to prevent T2DM.

Dietary strategies for the management of metabolic diseases have undergone a significant change. Previously, specific recommendations were provided for the intake of each nutrient in the diet. However, it was challenging to adapt to such complex diets. Healthy dietary patterns such as the Mediterranean diet (MedDiet) and Dietary Approaches to Stop Hypertension (DASH) are currently recommended to lower the risk of metabolic diseases. These are easy to follow and they can be applied to one’s diet to make it healthier [5]. MedDiet and DASH are basically plant-based diets that are rich in fruits, vegetables, whole grains, seeds, herbs, spices, and low-fat dairy foods [5]. The DASH diet emphasizes the consumption of lower quantities of sodium to prevent hypertension compared to MedDiet. Despite the fact that Asian diets are somewhat similar to the MedDiet and DASH, it is difficult to modify these diets to the Asian dietary pattern. In fact, given the uniqueness of the diet in each country in Asia, the dietary patterns need to be customized for each country to include healthier components to prevent T2DM or delay its progression. 

A traditional Korean balanced diet defined using a principal component analysis was observed to be inversely associated with a metabolic syndrome risk in the Health Examinees (HEXA) study in Korea [6]. A calcium- and vitamin C-rich diet characterized by the Korean Healthy Eating Index (KHEI) was also inversely associated with a metabolic syndrome (MetS) risk in the Korea National Health and Nutrition Examination Survey (KNHANES) 2013–2017 [7]. The KHEI reflects the Korean dietary patterns recommended by the Korean Disease Control and Prevention Agency (KDCA). The KHEI encompasses eight adequacy components and evaluates compliance with the ideal consumption of recommended foods such as fruit, vegetable, and milk and the intake of three foods that should be limited, such as sodium and saturated fatty acids. In addition, three balance components that assess the balance of energy intake have been included. However, there has been no research on which of these components predominantly contribute to the T2DM risk. In a systematic review based on prospective cohort studies and five different diet indices, namely, the alternate healthy eating index (AHEI), DASH, the dietary inflammatory index (DII), the healthy eating index (HEI), and the MedDiet, adherence to the HEI, AHEI, DASH, and the MedDiet was observed to decrease the incidence of cardiovascular disease (CVD) and T2DM [8]. However, these indexes are based on the diet in Western countries where meat consumption is high and grain consumption is low. In Asian countries, the intake of carbohydrates is high, and there is a relatively low intake of fatty meats. Additionally, a healthy diet in Asians may influence T2DM differently since the etiology of T2DM among Asians differs.

In Korea, Korean- and Western-style diets may influence T2DM incidences differently since the westernization of diets is considered to be the primary cause of a higher incidence of metabolic diseases, especially T2DM. The diet indexes for Korean- (KSD) and Western-style diets (WSD) have been generated by stratifying KHEI, and the modified KHEI (MKHEI) for KSD has been demonstrated to be inversely associated with abdominal obesity risk, while the MKHEI of WSD was not [9]. Accordingly, we hypothesized that KHEI and its components are associated with a lower T2DM risk and that the MKHEI for KSD and WSD is linked to an elevated T2DM risk. The hypothesis was examined using data from the KNHANES-VI (2013–2017).

## 2. Materials and Methods

### 2.1. Design and Data Collection

This study was conducted using data from the KNHANES-VI (2013–2017). The participants were aged 20–64 years and included using a rolling sampling design with a complex, stratified, multistage probability-cluster survey of a representative sample of the non-institutionalized civilian population in South Korea [10]. The KDCA and the Korean Ministry of Health and Welfare conducted the KNHANES with rigorous quality controls. The KNHANES is the representative data of the Korean population. The study was conducted in accordance with the Helsinki Declaration as revised in 2008 and approved by the Institutional Review Board of the KDCA (approval no. 2013-07CON-03-4C).

Participants aged 20–64 were included for analysis, and those who did not complete the demographic, health-related, and nutrition surveys were excluded [10]. Pregnant and perinatal women were also excluded. The total number of participants was 12,317. The participant’s ages, residences, regions, education, income, alcohol intake, energy intake, smoking status, marriage, and exercise status were obtained through interviews. Anthropometric measurements and biochemical test data were also collected. Height and weight were measured with the subjects wearing light clothing and no shoes. During the health interview, age was categorized into five groups, including 20–29, 30–39, 40–49, 50–59, and 60–64. Educational levels were stratified into ≤middle school, high school, and ≥university, and the monthly income levels were classified into four quartile groups. The area of residence was categorized into rural or city areas, and regions were classified into five regions according to the districts in Korea, as previously described [11]. 

The subjects’ smoking status was categorized as non-smokers, former smokers, and smokers, based on self-reporting as follows: a current smoker was defined as having smoked over 100 cigarettes in his/her lifetime, and a former smoker as having smoked no cigarettes during the last 6 months. Daily alcohol consumption was estimated by multiplying the average alcohol drinking frequency by the amount (in mL) consumed on a single occasion. Drinking status was categorized into none, mild (1–15 g), moderate (16–30 g), and heavy alcohol drinking (>30 g) per day. Regular exercise was defined as engaging in moderate physical activities of either ≥30 min at a time at least five times per week or ≥20 min at a time of vigorous physical activities at least three times per week. Moderate physical activity included carrying light objects, swimming slowly, playing doubles tennis or volleyball, and participating in occupational or recreational activities. The vigorous physical activity comprised running, climbing, cycling fast, swimming fast, playing football, basketball, squash, or singles tennis, jumping rope, occupational or recreational activities, and carrying heavy objects. 

### 2.2. Biochemical Tests

Blood was drawn into tubes with and without anticoagulants after 13–16 h of fasting. Total (TC) and high-density lipoprotein (HDL) cholesterols, triacylglycerol (TG), glucose, aspartate transaminase (AST), and alanine transaminase (ALT) were determined using an automatic analyzer (Hitachi 7600; Tokyo, Japan). The Friedewald equation was used to calculate low-density lipoprotein cholesterol (LDL) when serum TG concentration was less than 400 mg/dL [12]. Biochemical analyses were conducted at the Neodin Medical Institute, certified by the Korean Ministry of Health and Welfare.

T2DM in subjects was diagnosed based on the following criteria: ≥126 mg/dL fasting plasma glucose concentration, ≥6.5% HbA1c, or those taking hypoglycemic medication [13]. The number of participants with T2DM was 949 (442 for women and 507 for men), and that of non-T2DM was 11,368 (6989 for women and 4379 for men).

### 2.3. Determination of Dietary Quality and Nutritional Intakes by 24 h Recall and Semi-Quantitative Food Frequency Questionnaires (SQFFQ)

The 24 h recall method was used to estimate daily dietary intakes and to examine food types and amounts consumed during the previous 24 h through personal interviews by trained dieticians. Daily calorie and nutrient intake were calculated from the food intake obtained from the interviews using the computer-aided nutritional analysis program (CAN-Pro) 2.0 software developed by the Korean Nutrition Society.

The usual food intakes were estimated with a validated SQFFQ containing 116 food items that are commonly consumed by Koreans. The SQFFQ was developed and validated by the Ministry of Health and Welfare [14]. The food intake frequency of each participant was quantified using nine categories: never or seldom, once a month, two to three times a month, one to two times a week, three to four times a week, five to six times a week, once a day, twice a day, and three times or more every day [14]. The consumed foods were determined by half the portion size, one portion size, and double portion size. 

### 2.4. KHEI and MKHEI Scores for the KSD and WSD

KDCA designed KHEI to facilitate the assessment of the participants’ diet quality. It includes 12 adequacy components used to evaluate the suitable consumption of recommended foods such as fruit, vegetable, and milk; moderation components that evaluate the use of nutrients that may be harmful when used in excess, including saturated fatty acids, sugar, and sodium; ultra-processed food intake including fast foods and noodles; and balance components for assessing the balance of energy intake and the intake of 5 nutrients [12,15,16]. The detailed KHEI is presented in Supplementary Table S1. The KHEI of each participant was scored based on the SQFFQ.

The components related to the KSD were stratified, and the sum of the scores of the components was used as the MKEHI scores for the KSD. The MKEHI for the WSD was generated with the components for the WSD.

### 2.5. Statistical Analysis

Statistical analyses were performed using SAS software (version 9.4; SAS Institute, Cary, NC, USA) and statistical software for analyzing correlated data (SUDAAN, Release 11.0; Research Triangle Institute, Research Triangle Park, NC, USA) to incorporate sample weights and adjusted analyses for the complex sample design of the survey. Therefore, the results are representative of the free-living civilian population of Korea.

Descriptive statistics of categorical variables were determined using frequency distributions, and their statistical significance was analyzed using Chi-squared tests. Adjusted means and 95% confidence interval (CI) of the KHEI scores and the primary nutrient intakes were assessed using the ANCOVA test after covariate adjustment, including age, residence (rural or city), region, education, income, alcohol intake, energy intake, smoking status, marriage, and exercise.

Next, the adjusted odds ratios (ORs) and 95% CIs for T2DM according to the KHEI or MKHEI quartile score were examined using logistic regression analysis after covariate adjustment, as shown above. 

## 3. Results

### 3.1. Demographic Characteristics of the Participants

The proportion of men with T2DM was twice that of women. The proportion also increased with age, and the proportion of participants aged 60–64 with T2DM was 21.1% (Table 1). Participants in rural areas had T2DM more frequently than city dwellers. The number of T2DM participants with low education levels (<high school) was 3.5 times higher than those with a college education or more (Table 1). Income showed a trend similar to that of education. T2DM prevalence was lower among current smokers compared to non-smokers and past smokers (Table 1). The prevalence of T2DM was lowest in mild drinkers among all the drinking categories. However, regular exercise was not observed to significantly affect the prevalence of T2DM. Interestingly, married adults exhibited a significantly higher proportion of T2DM, 3.5 times higher than single adults (Table 1).

### 3.2. Anthropometric Measurements and Serum Glucose and Lipid Profiles According to Gender and T2DM

The serum glucose concentrations were much higher in the T2DM (151.4 in men and 149.7 mg/dL in women) than the non-T2DM group (95.3 in men and 92.2 mg/dL in women) for both genders (Table 2). The T2DM group had higher serum TC, LDL, and TG concentrations and lower HDL concentrations than the non-T2DM group for both genders (Table 2). The waist circumferences and BMI were higher in the T2DM group than in the non-T2DM group regardless of gender (Table 2).

### 3.3. KHEI Scores Based on SQFFQ According to Gender and T2DM Status

The components of KHEI comprise the adequate intake of essential nutrients, moderation in harmful nutrients and foods, and the balance of energy and beneficial nutrients. Each item was given a higher score when the corresponding food or nutrient intake was higher. Overall, the adequacy items were much lower in the T2DM versus the non-T2DM group (Table 3). Women with T2DM consumed significantly less fresh fruit and total fruit than those without T2DM, but men did not show significant difference of fruit intake between T2DM and non-T2DM groups. Men with T2DM consumed mixed grains significantly higher than those with non-T2DM (Table 3). Furthermore, T2DN women, versus non-T2DM women, had lower consumption scores for fish, meat, eggs, milk, and milk products. Overall, the moderation scores did not differ according to T2DM status or gender (Table 3). However, the noodle intake among the moderation items was higher in the T2DM women only. The overall scores of the balance items were lower in the T2DM than the non-T2DM group in women only. Among the balance items, vitamin C, carbohydrates, and fat percentages were lower in the T2DM than in the non-T2DM groups in women only (Table 3). The KHEI scores were only lower in the T2DM women (Table 3).

### 3.4. Nutrient Intake Using 24 h Recall According to Gender and T2DM Status

The daily energy intake was close to the dietary reference intake in both genders and it did not differ between the T2DM and non-T2DM groups. Carbohydrate, protein, and fat intakes were about 68 energy percent (En%), 13 En%, and 19 En%, respectively, in both genders. Among them, only the fat intake, including saturated and monounsaturated fatty acids, was higher in non-T2DM than T2DM (*p* = 0.018) in women. Furthermore, the micronutrient intake did not differ according to the T2DM status in men based on the 24 h recall (Table 4). However, in women, calcium, vitamin C, retinol, and vitamin B2 intakes were higher in the non-T2DM than in the T2DM group (Table 4).

### 3.5. Association of Total KHEI Score with T2DM Risk

**Figure 1:**
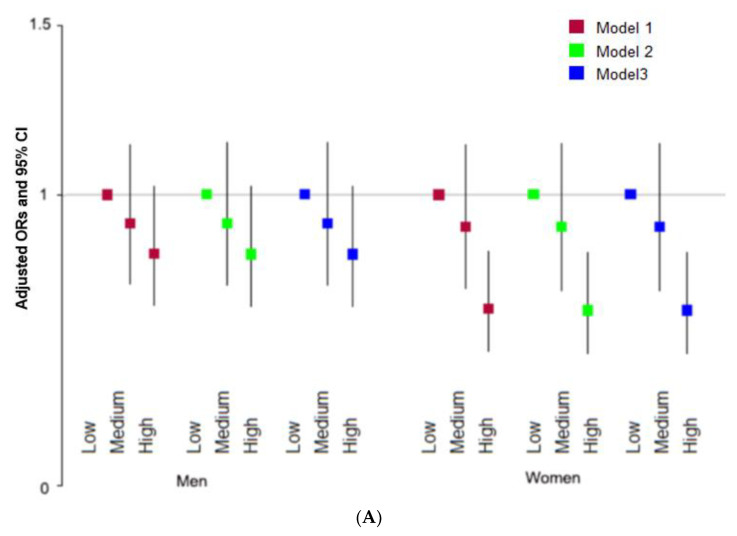

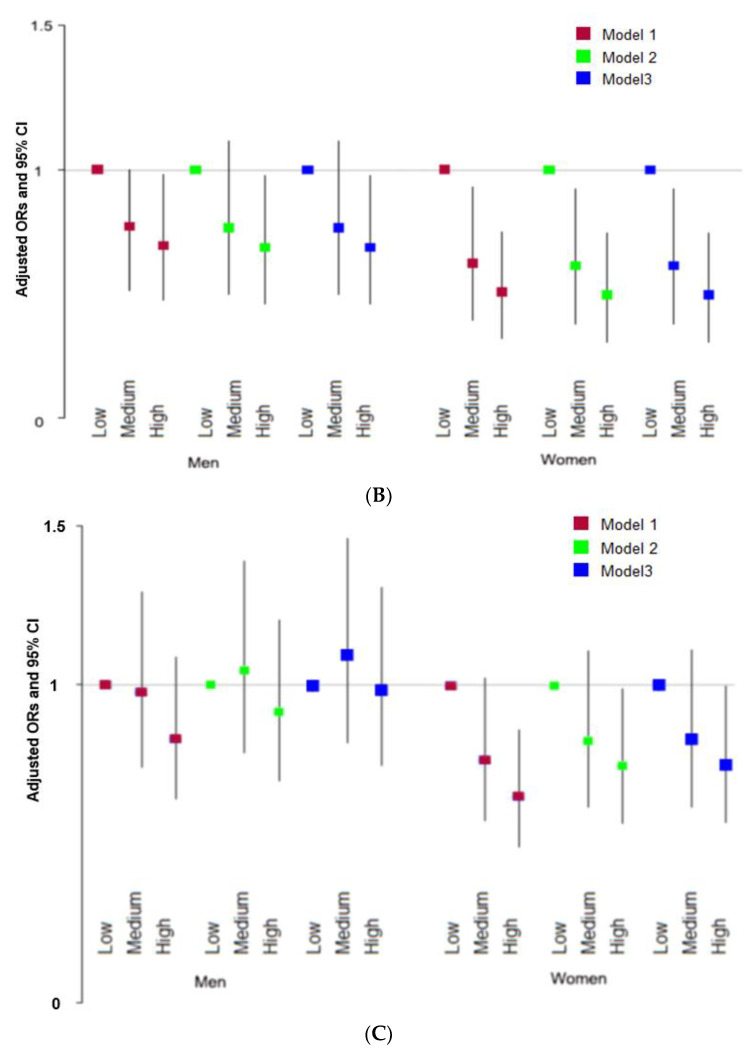
Association of the Korean healthy eating index (KHEI) scores with type 2 diabetes risk. (**A**) Adjusted odds ratio (ORs) of overall scores of KHEI and 95% confidence intervals (CI). (**B**) Adjusted ORs of adequacy scores of KHEI and 95% confidence intervals (CI). (**C**) Adjusted ORs of balance scores of KHEI and 95% confidence intervals (CI). ORs and 95% CI are represented with colored boxes and lines, respectively. Models were generated with different covariates, including age, gender, regions, and area of residence for model 1; covariates for model 1 plus education, income, marital status, and obesity for model 2; and covariates for model 2 plus energy intake, smoking, alcohol consumption, and regular exercise for model 3.

The total KHEI score was inversely associated with the T2DM risk only in women after adjusting for covariates (Figure 1A). Models were generated with different covariates, including age, gender, and regions as follows: model 1, residence area; model 2, covariates for model 1 plus education, income, marital status, and obesity; and model 3, covariates for model 2 plus energy intake, smoking, alcohol consumption, and regular exercise. In women, the adequacy and balance KHEI scores were inversely associated with the T2DM risk after adjusting for covariates in models 1, 2, and 3 (Figure 1B,C, respectively).

### 3.6. Association of MKHEI Scores for KSD and WSD with T2DM Risk

**Figure 2:**
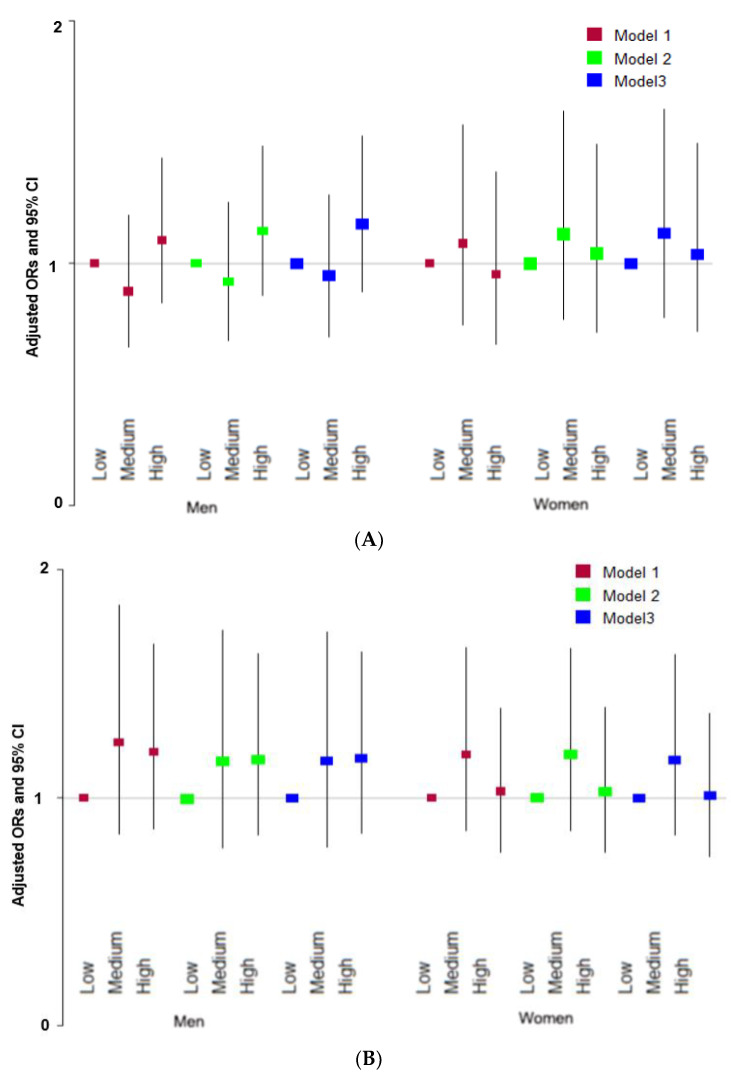
Association of the modified Korean healthy eating index (MKHEI) scores of Korean- and Western-style diets with type 2 diabetes risk. (**A**) Adjusted ORs of MKHEI scores of Korean-style diets; (**B**) Adjusted ORs of MKHEI scores of Western-style diets; ORs and 95% CI are represented with colored boxes and lines, respectively. Models were generated with different covariates, including age, gender, regions, and area of residence for model 1; covariates for model 1 plus energy intake, education, income, marital status, and obesity for model 2; and covariates for model 2 plus smoking, alcohol consumption, and regular exercise for model 3.

Neither the MKHEI scores for the KSD nor the WSD were not associated with T2DM risk regardless of gender after adjusting for covariates (Figure 2A,B).

## 4. Discussion

The HEI is a valuable diet quality index based on a 10-component system of five food groups, four nutrients, and scoring of the variety in food intake [17]. Each of the 10 components were scored on a scale of 0 to 10; to add up to 100 being the highest possible score. Higher HEI scores suggest a healthier diet. In each country, the HEI is modified to represent the country-specific diet pattern to arrive at a modified health eating index score [7,12]. KDCA has also generated a KHEI that is precisely aligned with the dietary guidelines for Korean [18]. While there is limited research on the relationship between KHEI and T2DM, a high KHEI score is known to be associated with a lower risk of developing T2DM. This study suggested that the overall KHEI and adequacy and balance component scores determined using an SQFFQ were significantly higher in the non-T2DM group than in the T2DM group only in women. Both KHEI and the 24 h recall results provided consistent nutrient intake results. The KHEI scores were inversely associated with T2DM only in women. However, only in women, the MKHEI scores for the KSD and WSD did not differ between the non-T2DM and T2DM groups. Thus, following Korean dietary guidelines is critical in preventing and/or delaying T2DM progression in women. KHEI analysis can be a helpful tool to identify which components of the Korean dietary guidelines are effective in lowering the T2DM risk among men and women.

Overall, the waist circumferences were greater in the T2DM group than in the non-T2DM group [19]. The prevalence of dyslipidemia was also elevated with T2DM, which is consistent with earlier studies [20,21]. Abdominal obesity related to increasing waist circumferences is linked to an elevated insulin resistance, which requires increased insulin secretion to maintain glucose homeostasis [19]. However, the insulin secretion capacity is lower in Asians than Caucasians, and T2DM incidences are quickly rising in Asians with high insulin resistance [19,22]. Therefore, adults with T2DM need to reduce their waist circumference and control their lipid profiles. T2DM incidence rates have demonstrated gender differences worldwide due to gender-specific differences in energy and glucose metabolism [19]. Although women generally have more body fat than men, more is stored in subcutaneous adipose tissue, whereas men preferably accumulate more visceral and ectopic fat [22]. Men are more insulin-resistant than women due to abdominal obesity [22]. In this study, along with waist circumferences, men had much higher serum triglyceride and lower HDL concentrations than women. The present study also revealed that the incidence of T2DM among men was twice that seen in women. However, women’s waist circumferences were lower than men’s in the T2DM group, suggesting that women may be susceptible to T2DM risk when abdominal obesity is induced.

The gender disparities in the KHEI are complex and multifactorial and are also influenced by factors such as socioeconomic status, education, and access to healthy foods, as seen in other countries [23]. Consistent with the present study, gender differences in the KHEI have been observed in earlier studies in other countries. Women generally have higher scores than men, indicating that the former have a better diet quality [23]. The differences in the KHEI between men and women are linked to gender differences in food preferences and health consciousness [24,25]. Women generally consume more fruits and vegetables, legumes, and whole foods but also consume more sugar and cake than men [26]. Men tend to have diets with higher fats and protein and consume more alcoholic beverages [26]. This study revealed that T2DM women had higher scores due to fruit, fish, meats, eggs, and milk intake in their diet than those without T2DM. However, a similar trend was not seen in men. The results indicated that higher scores associated with the intake of fruits, fish, and milk and proper amounts of meat and eggs lowered the risk of T2DM in adults. The scores associated with fruit, fish, and milk intake are consistent with previous studies [27,28]. However, the KHEI scores associated with meat intake seemed to be different from other studies. Red meats belong to the category of T2DM risk-increasing foods, and in one meta-analysis, their intake increased T2DM risk three-fold [29]. In the KHEI, the intakes of meats and eggs were 2–2.9 and 1.5–1.9 servings/d for men and women aged 19–64 years, respectively, and they were 1.5–1.9 and 1.5–2 servings/d for men and women aged ≥ 65 years, respectively. The results suggest that the meat intake might be optimal for Asians compared to the excessive meat consumption among Caucasians. Further studies are needed to stratify the types of meat based on their potential role in raising T2DM risk. Therefore, better adherence to KHEI in women may be related to a lower T2DM incidence than in men.

KHEI comprises the items for adequate food intake, moderation, and balance of nutrient intake. In the current study, the components of adequacy and balance, but not moderation, were associated with T2DM risk. The scores for the moderation items in T2DM and non-T2DM groups were high enough not to affect the T2DM risk. In the adequacy items, the scores of fresh fruits (≥1 serving/d) and total fruit intake (≥2 serving/d) were lower only in women in the T2DM group. However, the scores of total vegetables, including fermented vegetables (≥3 servings/d), were unaffected by the T2DM status. Previous studies have demonstrated that consuming fruits and vegetables lowers the risk of T2DM. A meta-analysis of 17 publications has shown that the overall effects of fruit and vegetable intakes are marginally inversely associated with T2DM risk [27]. Fruit intake has been shown to be non-linearly inversely associated with T2DM risk, and the optimal threshold of fruit intake is 250 g/day, and its intake decreases T2DM risk by about 13% [30,31]. However, a green leafy vegetable intake of up to 1.45 servings is almost linearly associated with T2DM risk [31]. In a 4-year longitudinal study, men consumed fewer fruits and vegetables than women (*p* < 0.001). Compared with high intakes, a low intake of vegetables by men resulted in a 62% greater risk of developing T2DM over 4 years after adjusting for age, education, BMI, smoking, alcohol, and physical activity [28]. Women show a similar trend of T2DM risk with a low green leafy vegetable intake without adjustments, but no significant increase after adjusting for age, education, BMI, smoking, alcohol, and physical activity [28]. The present study showed that fruit intake (≥100 g/d) was inversely associated with T2DM risk in women only, but vegetable intake lacked any association with T2DM for either gender. This lack of association of vegetable intake with T2DM risk might be related to the general high intake of vegetables in both the T2DM and non-T2DM groups (about five servings/d for men and three servings/d for women). An adequate vegetable intake, however, may not decrease T2DM risk unless the intake is less than or equal to two servings. Therefore, an adequate intake of fruits and vegetables is essential to reduce T2DM risk. 

Among the balance components, the percentages of carbohydrate and fat intakes based on the daily energy intake were significantly lower in T2DM than in the non-T2DM group in women only, suggesting that 55–65 energy percent of the energy derived from carbohydrates and 15–30 energy percent derived from fats were the optimal levels for decreasing the T2DM risk in women. The sources of carbohydrates and fats did not differ according to T2DM status in either gender, except for whole grain intake in men and noodle intake in women. The scores of whole grain intake were higher in the T2DM than the non-T2DM group in men only, reflecting that those with T2DM have a higher whole grain intake than those without T2DM, due to the cross-sectional design of this study. In a Chinese 11-year follow-up study, rice intake was not associated with an elevated T2DM risk. However, substituting noodles for one daily serving of rice was associated with T2DM risk [32]. Replacing one daily serving of rice with bread was not associated with T2DM risk [32]. However, another study failed to show an association between pasta intake and long-term T2DM risk [33]. The present study demonstrated that the scores of noodle intake were lower in the T2DM group than the non-T2DM group only in women, suggesting that adults consuming higher amounts of noodles had a higher T2DM incidence. Therefore, over one serving of noodles daily might adversely affect T2DM risk. 

This study is based on data from a large-scale, nationally representative survey that utilizes a complex sample survey design, making it a highly reliable and robust study. It was novel to demonstrate an increased T2DM risk among Korean women with an inadequate intake of fruits, milk, and fish, excessive quantities of meat and eggs, and an imbalance of vitamin C, carbohydrate, and fat intakes. The results provide important practical information for how the Asian population can lower their risk of T2DM due to their Westernized lifestyles, including diets. However, this study does have some limitations. First, the cross-sectional design shows associations but not cause-and-effect relationships. Second, the results could be skewed by bias in inaccurate self-reporting of dietary intake, physical activity, and smoking status. Third, the SQFFQ with 116 commonly consumed foods in Korea might not include some of the actual foods consumed, and this could lead to an underestimation of the food intake. Finally, the etiology of T2DM, such as the insulin secretion capacity and insulin resistance, was not determined, since the serum insulin concentrations were not measured. 

In summary, the overall KHEI score, as well as adequacy and balance scores among its components, were significantly higher in the non-T2DM group than in the T2DM group, but the effect was limited to women. Only in women were the KHEI scores inversely associated with T2DM. The score of mixed grain intake was higher in the non-T2DM than the T2DM group in men only. Consistent with the KHEI, the calcium, vitamin C, saturated and monounsaturated fatty acid, retinol, and vitamin B2 intakes derived from 24 h recall data were higher in the non-T2DM than the T2DM group in women only. However, the MKHEI scores for the KSD and the WSD did not differ between the non-T2DM and T2DM groups. Therefore, diet quality is an important determinant of T2DM risk regardless of the type of meal consumed, and the westernization of diets might not be the primary factor in increased T2DM incidence. Furthermore, high adequacy and balance component scores in the KHEI may effectively prevent and/or delay T2DM risk in women. Therefore, a Korean-style diet high in vitamin C, calcium, monounsaturated fatty acids, and retinol, while limiting the intake of fast foods and processed foods, can be recommended to control blood glucose concentrations in the general population. 

## Figures and Tables

**Table 1:** Distribution of study population by diabetes according to socioeconomic and lifestyle variables.

Parameters	Categories	T2DM (n = 949)	Non-T2DM (n = 11,368)	*p* Value *
Sex	Male	507 (8.9)	4379 (91.1)	<0.01
	Female	442 (5.2)	6989 (94.8)	
Age groups	20–29	14 (0.8)	1765 (99.2)	<0.01
	30–39	73 (2.8)	2787 (97.2)	
	40–49	191 (6.8)	2899 (93.2)	
	50–59	373 (12.3)	2778 (87.7)	
	60–64	298 (21.1)	1139 (78.9)	
Residence	Urban	746 (6.7)	9499 (93.3)	<0.01
	Rural	203 (8.7)	1869 (91.3)	
Region	Seoul	347 (6.8)	4296 (93.2)	<0.01
	Kyunggee-do	248 (5.7)	3424 (94.3)	
	Chungcheong-do	111 (7.8)	1187 (92.2)	
	Jeonlla-do	111 (8.1)	1209 (91.9)	
	Gyungsang-do	132 (8.9)	1252 (91.1)	
Education	Less than high school	333 (14.3)	1861 (85.7)	<0.01
	High school	324 (9.0)	3250 (91.0)	
	College	292 (4.1)	6257 (95.9)	
Income	1st Q	171 (12.7)	935 (87.3)	<0.01
	2nd Q	259 (7.8)	2712 (92.2)	
	3rd Q	244 (5.7)	3679 (94.3)	
	4th Q	271 (6.2)	4008 (93.8)	
Smoking status	Current smoker	480 (5.4)	7249 (94.6)	<0.01
	Past smoker	217 (9.6)	1839 (90.4)	
	Non-smoker	252 (9.0)	2280 (91.0)	
Drinking status	None	285 (9.2)	2702 (90.8)	<0.01
	Mild	412 (5.3)	6206 (94.7)	
	Moderate	105 (6.9)	1255 (93.1)	
	Severe	147 (10.6)	1205 (89.4)	
Regular exercise	Yes	415 (6.0)	5645 (94.0)	0.79
	No	534 (8.1)	5723 (91.9)	
Marriage	Yes	890 (8.5)	9142 (91.5)	<0.01
	No	57 (2.4)	2222 (97.6)	
Year	2013	250 (7.3)	3011 (92.7)	0.53
	2014	226 (6.9)	2754 (93.1)	
	2015	229 (6.4)	2722 (93.6)	
	2016	244 (7.4)	2881 (92.6)	

*: Chi-square test for each classification variable for diabetes.

**Table 2:** Adjusted mean ^¶^ and 95% CI of serum glucose concentration, lipid profiles, and BMI according to genders and diabetic condition after adjustments for covariates.

	Female (n = 7431)		*p* value	Male (n = 4886)		*p* Value *
	Diabetes (n = 442)	Normal (n = 6989)		Diabetes (n = 507)	Normal (n = 4379)	
Glucose	150 (144~155)	92.2 (91.9~92.4)	0.001	151 (146~157)	95.3 (94.9~95.6)	0.001
Chol	184 (179~188)	190 (189~1901)	0.001	186 (181~192)	191 (190~192)	0.001
HDL	50.4 (49.3~51.6)	55.9 (55.6~56.3)	0.001	44.3 (43.2~45.3)	47.9 (47.6~48.3)	0.001
LDL	104 (99.9~108)	113 (112~114)	0.001	95.5 (90.6~100)	111 (110~112)	0.001
TG	145 (135~155)	105 (103~107)	0.001	233 (212~255)	162 (157~166)	0.001
Waist	83.0 (81.9~84.2)	76.7 (76.4~76.9)	0.001	89.3 (88.2~90.3)	84.8 (84.5~85.1)	0.001
BMI	25.3 (24.8~25.8)	22.9 (22.8~23.0)	0.001	25.7 (25.3~26.1)	24.4 (24.3~24.6)	0.001

^¶^: adjusted by age, residences (rural or city), regions, education, income, alcohol intake, energy intake, smoking status, marriage, and exercise. Chol, total cholesterol; TG, triglyceride; and BMI, body mass index. *: *p*-value S_waite ChiSq.

**Table 3:** Adjusted means ^¶^ and 95% CI of KHEI score according to genders and diabetes after adjustments for covariates.

	Classification	Female (n = 7431)			Male (n = 4886)		
		T2DM (n = 442)	Non-T2DM (n = 6989)	*p* Value	T2DM (n = 507)	Non-T2DM (n = 4379)	*p* Value *
Adequacy	Have breakfast	6.878 (6.48~7.276)	7.023 (6.913~7.133)	0.475	6.911 (6.547~7.275)	6.697 (6.565~6.828)	0.268
	Mixed grains intake	4.283 (4.112~4.453)	4.119 (4.069~4.169)	0.065	4.171 (4.004~4.338)	3.748 (3.679~3.817)	0.001
	Fresh fruit intake	3.469 (3.286~3.652)	3.791 (3.747~3.835)	0.001	2.298 (2.111~2.485)	2.486 (2.422~2.549)	0.054
	Total fruit intake	3.393 (3.211~3.576)	3.684 (3.638~3.73)	0.002	2.065 (1.878~2.251)	2.215 (2.152~2.277)	0.126
	Vegetable intake, excluding kimchi and pickled vegetables	4.916 (4.876~4.956)	4.896 (4.882~4.91)	0.345	4.755 (4.683~4.828)	4.827 (4.803~4.851)	0.057
	Fermented vegetables, kimchi, and pickled vegetables	4.46 (4.321~4.598)	4.445 (4.411~4.478)	0.833	4.221 (4.069~4.372)	4.281 (4.234~4.328)	0.434
	Seaweed intake	1.426 (1.223~1.629)	1.507 (1.454~1.56)	0.445	0.78 (0.628~0.933)	0.814 (0.768~0.859)	0.676
	Fish intake	1.19 (0.998~1.382)	1.581 (1.523~1.638)	0.001	1.501 (1.306~1.696)	1.555 (1.485~1.625)	0.606
	Meat and eggs	2.416 (2.205~2.627)	2.691 (2.639~2.743)	0.012	2.867 (2.676~3.059)	2.95 (2.895~3.004)	0.424
	Beans, including fermented beans	1.24 (1.035~1.445)	1.248 (1.198~1.298)	0.938	1.471 (1.273~1.669)	1.354 (1.284~1.424)	0.284
	Milk and milk products intake	3.235 (2.806~3.665)	3.704 (3.584~3.823)	0.039	3.238 (2.842~3.634)	3.372 (3.236~3.507)	0.528
	Nuts	0.384 (0.197~0.571)	0.414 (0.371~0.458)	0.756	0.351 (0.161~0.542)	0.464 (0.404~0.524)	0.291
	KHEI_A_All	37.19 (36.04~38.35)	38.98 (38.65~39.31)	0.002	34.52 (33.47~35.58)	34.59 (34.18~34.99)	0.91
Moderation	Percentage of energy from saturated fatty acids	9.433 (9.334~9.533)	9.403 (9.372~9.435)	0.549	9.486 (9.397~9.576)	9.454 (9.419~9.489)	0.488
	Percentage of energy from polyunsaturated fatty acids	4.701 (4.513~4.888)	4.691 (4.639~4.742)	0.915	4.323 (4.206~4.44)	4.327 (4.283~4.372)	0.941
	Sodium intake	5.301 (4.907~5.695)	5.258 (5.154~5.361)	0.831	3.894 (3.527~4.261)	3.84 (3.718~3.963)	0.786
	Percentage of energy from sweets and beverages	3.738 (3.54~3.935)	3.643 (3.583~3.702)	0.361	4.086 (3.88~4.292)	3.983 (3.918~4.049)	0.353
	Fast food intake	3.78 (3.663~3.897)	3.848 (3.819~3.876)	0.273	3.49 (3.356~3.623)	3.483 (3.443~3.524)	0.931
	Noodle intake	3.405 (3.372~3.438)	3.609 (3.483~3.736)	0.002	3.301 (3.256~3.345)	3.338 (3.192~3.484)	0.628
	KHEI_M_All	30.40 (29.83~30.97)	30.14 (29.98~30.30)	0.382	28.44 (27.89~28.99)	28.34 (28.15~28.53)	0.724
Balance	Energy intake	3.847 (3.668~4.025)	3.951 (3.902~4.000)	0.269	3.813 (3.623~4.002)	3.954 (3.89~4.017)	0.162
	V-C intake	2.77 (2.536~3.005)	3.107 (3.042~3.172)	0.005	2.384 (2.163~2.605)	2.581 (2.501~2.661)	0.093
	Fiber intake	3.575 (3.418~3.732)	3.614 (3.573~3.655)	0.626	3.773 (3.633~3.914)	3.724 (3.675~3.774)	0.514
	Ca intake	1.07 (0.895~1.245)	1.138 (1.087~1.189)	0.451	1.53 (1.342~1.719)	1.572 (1.504~1.64)	0.682
	Percentage of energy from carbohydrates	3.021 (2.833~3.21)	3.212 (3.165~3.26)	0.057	3.192 (2.999~3.384)	3.279 (3.218~3.339)	0.399
	Percentage of energy intake from fats	3.862 (3.68~4.044)	4.141 (4.105~4.178)	0.041	3.906 (3.736~4.077)	4.025 (3.977~4.072)	0.202
	KHEI_B_All	17.43 (16.79~18.06)	18.29 (18.11~18.46)	0.009	17.85 (17.18~18.51)	18.33 (18.12~18.55)	0.164
KHEI_All_Score		85.01 (83.5~86.5)	87.40 (86.99~87.82)	0.001	80.81 (79.47~82.15)	81.26 (80.77~81.74)	0.528

^¶^: adjusted by age, residences (rural or city), regions, education, income, alcohol intake, energy intake, carbohydrate intake, smoking status, marriage, and exercise; *: *p*-value S_weighted ChiSq.

**Table 4:** Adjusted mean ^¶^ and 95% confidence intervals of nutrient intake measured by 24 h recall according to genders and diabetic condition after adjustments for covariates.

	Female (n = 7431)			Male (n = 4886)		
	T2DM (n = 442)	Non-T2DM (n = 6989)	*p* Value	T2DM (n = 507)	Non-T2DM (n = 4379)	*p* Value *
Energy (kcal)	1772 (1706~1839)	1813 (1795~1832)	0.224	2433 (2343~2523)	2402 (2373~2430)	0.51
Fat (En%)	18.9 (18.3~19.4)	19.7 (19.4~19.7)	0.018	19.5 (19.0~20.1)	19.6 (19.4~19.8)	0.233
Protein (En%)	13.3 (13.1~13.6)	13.5 (13.5~13.6)	0.124	13.8 (13.5~14.0)	13.6 (13.6~13.7)	0.946
CHO (En%)	67.8 (67.1~68.6)	66.8 (66.6~67.0)	0.133	66.7 (66.9~67.5)	66.8 (66.6~67.1)	0.501
Fiber (g)	20.0 (19.0~20.9)	20.2 (20.0~20.5)	0.548	21.9 (20.9~23.0)	21.2 (20.9~21.6)	0.212
Calcium (mg)	442.9 (422.9~462.9)	466.0 (459.7~472.4)	0.026	525.0 (499.3~550)	524.1 (515.6~532.7)	0.972
Iron (mg)	12.9 (12.4~13.5)	13.113 (13.0~13.3)	0.578	15.6 (14.8~16.3)	15.1 (14.9~15.3)	0.236
Vitamin C (mg)	113.1 (103.3~122.8)	124.1 (121.5~126.7)	0.029	100.6 (93.4~107.8)	105.6 (102.9~108.4)	0.15
SFA (En%)	5.71 (5.52~5.9)	5.97 (5.92~6.02)	0.009	5.72 (5.5~5.9)	58.6 (5.8~5.92)	0.143
MUFA (En%)	6.01 (5.8~6.21)	6.26 (6.21~6.32)	0.017	6.03 (5.81~6.24)	6.14 (6.08~6.21)	0.312
PUFA (En%)	5.5 (5.3~5.7)	5.58 (5.53~5.63)	0.45	4.96 (4.8~5.12)	5.01 (4.96~5.06)	0.574
N3-FA (En%)	1.23 (1.16~1.31)	1.28 (1.26~1.30)	0.198	1.47 (1.39~1.55)	1.47 (1.45~1.50)	0.916
N6-FA (En%)	9.00 (8.46~9.54)	9.24 (9.09~9.38)	0.398	11.3 (10.6~11.9)	11.2 (10.9~11.4)	0.78
Phosphate (mg)	923.2 (883.7~962.7)	960.7 (949.3~972.1)	0.063	1154 (1105~1203)	1136 (1120~1152)	0.478
Sodium (mg)	3050 (2901~3200)	3101 (3058~3145)	0.507	3878 (3682~4075)	3797 (3731~3862)	0.427
Potassium (mg)	2649 (2510~2788)	2755 (2720~2792)	0.135	3020 (2887~3154)	2995 (2948~3041)	0.713
Vitamin A	614.8 (574.7~654.8)	623.0 (613.4~632.6)	0.091	660.0 (620.5~699)	647.3 (635.5~659.1)	0.533
Carotenoids (µg)	3079 (2856~3301)	3075 (3024~3126)	0.975	3235 (3031~3440)	3152 (3091~3214)	0.431
Retinol (µg)	80.8 (75.3~86.4)	88.4 (86.9~90.0)	0.008	97.5 (91.5~103.4)	98.3 (96.3~100.3)	0.779
Vitamin B1 (mg)	1.70 (1.63~1.77)	1.74 (1.72~1.76)	0.308	2.13 (2.40~2.2)	2.10 (2.07~2.13)	0.497
Vitamin B2 (mg)	1.22 (1.16~1.28)	1.29 (1.27~1.30)	0.025	1.53 (1.45~1.61)	1.52 (1.49~1.54)	0.803
Niacin (mg)	12.3(11.7~12.8)	12.7 (12.5~12.8)	0.18	15.9 (15.2~16.6)	15.5 (15.2~15.7)	0.263

^¶^: adjusted by age, residences (rural or city), regions, education, income, alcohol intake, energy intake, carbohydrate intake, smoking status, marriage, and exercise. En%, energy percent; SFA, saturated fatty acids; MUFA, monounsaturated fatty acids; PUFA, polyunsaturated fatty acids; N3-FA, n-3 polyunsaturated fatty acids; and N6-FA, n-6 polyunsaturated fatty acid. *: *p*-value S_waite ChiSq.

## Data Availability

The raw data involved in this study will be available by the authors to any qualified researcher.

## Supplementary Materials

The following supporting information can be downloaded at: https://www.mdpi.com/article/10.3390/nu15071741/s1, Table S1: Components of and scoring standards for modified Korean Healthy Eating Index.
